# How Should Stressors Be Examined in Teachers? Answering Questions about Dimensionality, Generalizability and Predictive Effects Using the Multicontext Stressors Scale

**DOI:** 10.3390/ijerph16183388

**Published:** 2019-09-12

**Authors:** Ángel Abós, Javier Sevil-Serrano, Lisa E. Kim, Robert M. Klassen, Luis García-González

**Affiliations:** 1 Faculty of Health and Sport Sciences, Department of Didactics of the Musical, Plastic and Corporal Expression, University of Zaragoza, Huesca 22001, Spain; 2 Department of Education, University of York, York YO10 5DD, UK

**Keywords:** teacher stressors, Multicontext Stressors Scale, measurement invariance, self-determination theory, basic psychological needs, burnout, anxiety, depression, job satisfaction, intention to quit

## Abstract

Using the Multicontext Stressors Scale (MSS), this study investigates which factorial structure should be used to measure teacher stressors, and the extent to which this factorial structure of MSS remains invariant across gender. Subsequently, grounded in self-determination theory, the present study also examines the extent to which stressors may differentially predict teachers’ psychological functioning. Participants were 584 (*M*_age_ = 45.04; *SD* = 8.97) secondary school teachers. Goodness-of-fit indices and estimated parameters of the models, together with latent correlations between stressors, offered support for the six-factor structure, whereas the opposite was true for the one-factor structure of the MSS. Results also supported gender invariance of the MSS. Predictive findings showed that student misbehavior, lack of shared decision-making, and workload stressors are negatively related to basic psychological needs. Likewise, results noted the important role of basic psychological needs to reach optimal teachers’ psychological functioning. The results are discussed, arguing the importance of assessing and analyzing teacher stressors using a multifactorial and invariant scale. From a more practical approach, it seems important for school leaders to be especially vigilant about all stressors. Nonetheless, if they desire to prevent detrimental psychological functioning in teachers, special attention should be placed on stressors related to student misbehavior, lack of shared decision-making, and workload.

## 1. Introduction

International research has shown that teachers perceive their job as a highly stressful occupation [1,2,3]. Teacher stress is frequently defined as the experience of unpleasant emotions resulting from multifaceted aspects of the teaching occupation [4,5]. According to this definition, considerable research has been conducted to identify the main job stressors in the teaching context. Student misbehavior, lack of recognition, student diversity, lack of decision-making, workload, and conflicts with parents, colleagues, and educational administration are some of the most commonly reported stressors in both qualitative and quantitative research [2,6,7]. Similarly, a large number of studies have shown the relationship between these stressful teaching conditions and a broad range of negative psychological outcomes (e.g., burnout, anxiety, depression) [6,8], which may have negative implications for the quality of education (e.g., low students’ motivation) [9,10].

Nonetheless, although the identification of stressors and some associations between teacher stressors and maladaptive outcomes (e.g., burnout, anxiety, depression, etc.) have been well-documented, some questions related to the dimensionality of stressors, cross-gender generalizability, or the examination of its effects following theoretical frameworks, still remain open. Aiming to fill these gaps, the present study relies on the Multicontext Stressors Scale (MSS) [6], a questionnaire measuring the most common stressors of the teaching profession [7]. Using the MSS, this study extends past research by investigating which type of factorial structure should be used to measure teacher stressors, as well as by testing its measurement invariance across gender. From a more practical point of view, grounded in self-determination theory (SDT) [11,12,13], this study also aims to know the extent to which stressors may differentially predict teachers’ psychological functioning, a topic barely explored to date.

### 1.1. Multicontext Stressors Scale (MSS): Gaps in Factorial Structure and Invariance

Teacher stressors are not purely static but they are factors with a dynamic nature that may vary depending on the changes that the educational system experiences [14]. For example, the social, political, and cultural changes that have been occurring in Spain over the last years (e.g., implementation of several education laws, and growing immigration over the past 15 years) have produced vast changes in the educational system to respond to these challenges. Keeping this in mind, the items and the factorial structure of the Stressor Multilevel Context Scale were modified and validated again [14], giving rise to the MSS [6]. As a result, six potential stressors in the school environment (31 items) were identified, accounting for 70.86% of the explained variance. The stressors are: student misbehavior, lack of shared decision-making, ambiguous demands from administration, student diversity, workload, and insufficient parent involvement. Despite the adequate fit indices [15], and relatively high reliability shown by the six factors (i.e., Cronbach’s αs ranged from 0.81 to 0.93), some inconsistencies in this factorial structure and cross-gender generalizability were not resolved.

With regard to the factorial structure, whereas the six-factor structure was properly examined using exploratory and confirmatory procedures [16], the one-factor structure (i.e., single common stressor) was not tested. More specifically, in the validation study of the MSS a second-order factor, which represented the six teacher stressors, was introduced into the predictive model to explain the relationship between stressors and burnout [6]. However, there was no evidence that the use of a single common stressor was adequate, which resulted in a biased model of the nominal validity of the MSS. Indeed, past research has reported moderate to low correlations (i.e., *r* = 0.15 to 0.45; *M* = 0.35) between the six teacher stressors of the MSS [15], as well as inconsistent association patterns between different sources of stress and teachers’ outcomes [17,18], offering little support for this assumed one-factor structure. For these reasons, it seems fundamental to undertake an in-depth study of the thoroughness of using single-factor structures to assess teacher stressors.

As regards measurement invariance across gender, it is well-known that a key prerequisite to guarantee an adequate psychometric validation of a scale is to demonstrate the extent to which the psychometric properties found in a sample can be generalized to other subgroups [19]. In this sense, while a large body of studies have shown that some sociodemographic factors, such as gender, may affect how a teacher perceives the stressors, measurement invariance has not been tested in any of the stressor scales used to date, including the MSS [6,15]. To illustrate, a recent research showed that female teachers perceive higher levels of workload but lower levels of value dissonance [20]. Similarly, Betoret and Artiga [15] found that, using the MSS with Spanish schoolteachers, there was a positive correlation between female teachers and the perception of some potential stressors such as student misbehavior, student diversity, and insufficient parent involvement. In this sense, before adding new evidence to the literature on teacher stressors, a rigorous examination of its complete measurement invariance across gender seems required.

### 1.2. Teacher Stressors and Psychological Functioning: The Role of Self-determination Theory

Within the SDT framework [11,12,13], individuals are presumed to have to satisfy three basic psychological needs (BPNs) to optimally develop and function. The three BPNs are conceptualized as autonomy, competence and relatedness [12,13]. In a teaching context, teachers experience a high sense of autonomy when they are able to determine their own actions and assume responsibility for actions concerning school development and the teaching process [21]. Teachers experience a high sense of competence when they feel that they are able to develop their abilities and achieve desired goals [22]. Finally, teachers experience a high sense of relatedness when they can establish close and positive relationships with their social environment (e.g., fellow teachers, principals, or students), and feel mutual respect [23]. A vast body of research underpins the significant role of satisfying the three BPNs for an adequate psychological functioning of teachers at work [24]. To illustrate, past studies in teaching settings have shown positive relationships between BPNs and job satisfaction [25,26], and negative relationships with adverse outcomes such as burnout [27], anxiety [28], depression [29], and intention to quit the job [30].

On the other hand, if teachers’ BPN satisfaction is positively related to higher psychological functioning, it is crucial to explore the roots that may affect the fulfillment of these basic needs. SDT states that the satisfaction of the three BPNs largely depends on a person’s social environment [11,12,13]. In this vein, the stressful teaching conditions that take place in teachers’ work environments may differentially affect their satisfaction of autonomy, competence, and relatedness [11,12,13]. However, there is a paucity of studies to date that have directly explored the associations between teacher stressors and satisfaction of the three BPNs. To our knowledge, only one study has examined the relationships between stressful teaching conditions and the BPNs in a sample comprised entirely of teachers [31]. In the referred study, different sources of stress were grouped together to create two one-factor variables (i.e., stressors inside and outside class). Both of these general stress factors, inside and outside class, showed negative relationships with the needs for competence and relatedness. However, the need for autonomy was not measured, which could have biased the associations between the stressors and BPNs [31]. Another study with employees (58% of whom were teachers) found that workload and ambiguity (i.e., representing lack of information to perform the tasks) were negatively related to autonomy and competence, respectively [32]. Yet, no relationships were found between any stressors and the need for relatedness. Although these studies show only partial associations between some stressful teaching conditions and BPN satisfaction, they suggest that the different sources of stress could impact teachers’ BPN satisfaction differently. Hence, more research considering other potential teacher stressors (e.g., student misbehavior, lack of shared decision-making, student diversity, among others) and the three BPNs seems necessary to attain a better understanding of the role of teacher stressors in their psychological functioning.

### 1.3. The Present Research

To overcome the abovementioned limitations of the MSS [6,15], and considering recent studies that call for a greater understanding of teacher stressors [17,18], the first aim of the study was to investigate the factorial structure of the MSS, as well as to analyze the extent to which the factorial structure of MSS ratings remains invariant across samples of male and female teachers. We expected to find sufficient statistical evidence to retain the six-factor structure rather than the one-factor structure of the MSS, and we also expected this scale to remain invariant regardless of the teachers’ gender.

Subsequently, the present study also aimed to investigate if the six stressors of the MSS have different effects on teachers’ psychological functioning. More precisely, the second aim of the present study was to examine the relationships between the six stressors of the MSS and the three basic needs, and the relationships between the three BPNs and job satisfaction, burnout, anxiety, depression, and intention to quit the job. Based on the tenets of SDT [11,12,13] and previous studies [31,32] we expected to find negative relationships between both variables. Yet, considering the scarce research conducted on this topic, our hypotheses should be tentative. Finally, we expected to find positive relationships between the three BPNs and job satisfaction, while negative relationships between BPNs and burnout, anxiety, depression, and intention to quit the job were postulated.

## 2. Materials and Methods

### 2.1. Participants and Procedures

As the target population was secondary school teachers, we contacted all in-service teachers (i.e., 7418) working in a Spanish region (i.e., Aragon) during the 2014/2015 academic year. The response rate was 8%, resulting in an intentional study sample of 584 Spanish secondary school teachers from 106 secondary schools (81 state schools, 25 non-state schools). Importantly, after the first data recruitment, no attempt was made to increase the sample using alternative methods. The mean age was 45.04 years (*SD* = 8.97), and they all taught in mixed secondary schools. The study sample included male (43%) and female (57%) teachers, which is equal to the proportion of male and female secondary school teachers in the region of Aragon (i.e., 3213 (43.1%) were males; 4186 (56.9%) were females). Likewise, 71% of the teachers worked in state schools (i.e., 415) whereas the 29% worked in non-state schools (i.e., 169), which also is equal to the proportion of state and non-state school teachers in the region of Aragon (i.e., 5279 (71.1%) worked in state schools; 2138 (29.9%) worked in non-state schools). These statistics were facilitated by the Spanish Ministry of Education and Vocational Training (http://www.educacionyfp.gob.es/).

Data were collected via an online questionnaire. Secondary school teachers received an e-mail with study access details, a brief explanation of the goals of the study, and the lead researcher’s contact details. The secondary school teachers’ contact information (i.e., e-mail) was obtained through the Educational Administration of the region of Aragon. The deadline to complete and submit the questionnaire was 30 days. The online questionnaire was designed to avoid missing values, ensuring that the responses were submitted only if completed until the end. Participation was voluntary and the confidentiality of the participants’ responses was guaranteed. Ethical approval for this study was obtained from the Ethics Committee for Clinical Research of Aragon (CEICA; PI15/0283).

### 2.2. Measures

#### 2.2.1. Multicontext Stressors Scale (MSS)

Teacher stressors were measured using the Spanish version of the Multicontext Stressors Scale (MSS) [6]. This scale starts with the stem, “Indicate to what extent the elements or conditions listed below make it difficult to fulfill the learning objectives with students”, and includes 31 items assessing student misbehavior (seven items; e.g., “Students’ lack of interest”, α = 0.89), lack of shared decision-making (five items; e.g., “The lack of autonomy to make my own decision”, α = 0.87), ambiguous demands from administration (six items; e.g., “The frequent changes to the study curriculum”, α = 0.94), student diversity (five items; e.g., “The cultural and racial diversity among students”, α = 0.87), workload (four items; e.g., “Excessive academic load”, α = 0.88) and insufficient parent involvement (four items; e.g., “Parents’ collaboration is insufficient”, α = 0.85). Teachers’ responses were registered on a 4-point Likert-type scale ranging from 0 (“they do not interfere with me in the least”) to 3 (“they interfere with me a great deal”). The six factor-structure has shown adequate psychometric properties and reliability (α ranging from 0.81 and 0.93) in previous studies with secondary school teachers [15].

#### 2.2.2. Basic Psychological Need Satisfaction

Teachers’ BPN satisfaction was measured using the Spanish version of the Basic Psychological Needs at Work Scale (BPNWS) [27]. This scale includes 12 items assessing autonomy satisfaction (four items; e.g., “I can use my judgment when solving work-related problems” α = 0.84), competence satisfaction (four items; e.g., “I succeed in my work” α = 0.84) and relatedness satisfaction (four items; e.g., “When I’m with the people from my work environment, I feel I am a friend to them” α = 0.90). Teachers’ responses were provided on a 6-point Likert-type scale ranging from 1 (“strongly disagree”) to 6 (“strongly agree”). This scale has shown adequate psychometric properties and reliability (α ranging from 0.83 and 0.86) in prior research with teachers [33]. In the present study, a CFA was conducted showing adequate goodness-of-fit (χ^2^/df = 4.11, *p* < 0.001; RMSEA = 0.071; CFI = 0.992; TLI = 0.990).

#### 2.2.3. Teacher Burnout

Teacher burnout was measured using the Spanish short-version of the Burnout Clinical Subtype Questionnaire (BCSQ-12) [34]. This scale includes 12 items assessing overload (four items; e.g., “I neglect my personal life when I pursue important achievements in my work”, α = 0.88), lack of development (four items; e.g., “I would like to be doing another job where I can better develop my talents”, α = 0.90) and neglect (four items; e.g., “When the effort I invest in work is not enough, I give in”, α = 0.89). For parsimony reasons, the SEM analyses were performed based on the composite score for burnout (i.e., 12 items, α = 0.85) rather than on the separate subtypes of overload, lack of development and neglect. Responses were registered on a 7-point Likert-type scale ranging from 1 (“strongly disagree”) to 7 (“strongly agree”). This questionnaire has shown adequate psychometric properties and reliability (α ranging from 0.88 and 0.90) in previous research with secondary school teachers [35]. In the current study, a CFA was conducted showing adequate goodness-of-fit (χ^2^/df = 2.08, *p* < 0.001; RMSEA = 0.043; CFI = 0.978; TLI = 0.971).

#### 2.2.4. Job Satisfaction

Teacher job satisfaction was measured using a Spanish translation of the Teacher Job Satisfaction Scale (TJSS) [36]. This four-item scale is comprised of a single factor (e.g., "When I get up in the morning, I look forward to going to work", α = 0.89). Teachers’ responses were provided on a 6-point Likert-type scale from 1 (“strongly disagree”) to 6 (“strongly agree”). This scale has shown adequate psychometric properties and reliability (α = 0.90) in previous studies with teachers [37]. In the present study, a CFA was performed showing adequate goodness-of-fit (χ^2^/df = 2.01, *p* < 0.001; RMSEA = 0.031; CFI = 0.992; TLI = 0.949).

#### 2.2.5. Anxiety and Depression

Anxiety and depression were measured using the Spanish version of the Hospital Anxiety and Depression Scale [38] This scale includes 14 items and taps into anxiety (seven items; e.g., “I feel restless as I have to be on the move”, α = 0.83) and depression (seven items; e.g., “I feel as if I am slowed down”, α = 0.81). Teachers’ responses were provided on a 4-point Likert-type scale from 0 (“strongly disagree”) to 3 (“strongly agree”). This scale has shown adequate psychometric properties in previous studies with teachers [39]. In the current study, a CFA was conducted showing adequate goodness-of-fit (χ^2^/df = 3.42, *p* < 0.001; RMSEA = 0.064; CFI = 0.921; TLI = 0.900).

#### 2.2.6. Intention to Quit the Job

Consistent with previous studies on teachers [40], participants were asked whether they had thought about quitting their jobs with the question, "Have you ever had thoughts of leaving your job as a teacher?”. The question was dichotomous (i.e., yes or no answer).

### 2.3. Data Analysis

First, the descriptive statistics and Pearson’s correlations between items of the MSS were calculated using SPSS 20.0 (IBM SPSS Inc., Chicago, IL, USA). Second, the models (i.e., CFAs and structural equation modeling -SEM-) were conducted using Mplus 7.3 (Muthén & Muthén, Los Angeles, LA, USA) with a robust maximum likelihood (MLR) estimator. Third, tests of measurement invariance across gender of the retained model were then examined in the following sequence [19]: (1) measurement invariance of the same pattern of free/fixed parameters (i.e., configural model); (2) measurement invariance of factor loadings/cross-loadings (i.e., weak model); (3) measurement invariance of factor loadings/cross-loadings, and intercepts (i.e., strong model); (4) measurement invariance of factor loadings/cross-loadings, intercepts, and uniquenesses (i.e., strict model) (5) measurement invariance of factor loadings/cross-loadings, intercepts, uniquenesses, and latent variances-covariances (i.e., latent variance-covariance model); and (6) measurement invariance of factor loadings/cross-loadings, intercepts, uniquenesses, latent variances-covariances, and latent means (i.e., latent means model). The first four tests of measurement invariance explored the presence of different biases, and provided sufficient evidence to state that the measurement properties of a questionnaire were equal across subgroups [41]. Further, the last two tests of measurement invariance may be helpful to identify the presence of significant and unbiased group differences taking place at the level of the latent variances, covariances, and means [41]. Finally, in order to inspect the associations between teacher stressors and their psychological functioning, a latent correlation analysis was conducted through the addition of CFA factors representing the three BPNs, job satisfaction, burnout, anxiety, depression, intention to quit the job, and gender. In addition, based on the SDT sequence, a SEM was estimated by once again using the retained solution and adding the same latent CFA factors as in the previous correlation analysis. The standardized regression weights (β) and explained variance (*R*^2^) were reported.

The different models were inspected through the following goodness-of-fit indices: comparative fit index (CFI), the Tucker-Lewis index (TLI), the root mean square error of approximation (RMSEA) with its 90% confidence interval. According to typical interpretation guidelines [42], values of more than 0.90 and 0.95 for the CFI and TLI, respectively, indicate adequate and excellent fit indices, while values of 0.08 and 0.06 or less for RMSEA, are considered as adequate and excellent fit indices. In addition, the Akaike information criteria (AIC), the Bayesian information criteria (BIC), and the sample-size adjusted BIC (ABIC) were also used to compare the CFA models. Lower values for AIC, BIC and ABIC suggest a better fitting model. The measurement invariance models were inspected comparing each test with its previous model by considering the following modifications (Δ): Higher decreases of 0.010 in CFI and TLI, and higher increases of 0.015 in RMSEA indicate a lack of invariance across gender [43].

## 3. Results

### 3.1. Factorial Structure and Invariance of the Multicontext Stressors Scale

The descriptive statistics and Pearson’s correlations for teachers’ responses to the MSS items are reported in Table 1. Overall, item correlations showed significant and strong relationships between the items of the same factor, while associations with the items of the other factors were moderate to low.

Apparently, the below results of item correlations display some indications of the need for a multifactor structure to evaluate teacher stressors. However, to obtain more rigorous evidence on this issue, a one-factor CFA model, a six-factor CFA model and, a second-order CFA model of the MSS were systematically conducted and compared. The goodness-of-fit statistics of the three measurement models estimated are reported in Table 2.

These results indicated that the fit of the one-factor CFA model, in which all the items were directly loaded on a single stressor, fell below acceptable values according to the CFI and the TLI (<0.900), as well as the RMSEA (≥0.080). In contrast, the six-factor CFA model, in which each item was loaded on its theoretical factor, fell within the range of acceptable values according to the CFI and the TLI (≥0.900), and excellent values according to the RMSEA (≤0.060). Finally, the second-order factor CFA model, in which each item was loaded on its theoretical factor, and hierarchically on a single common stressor, too, although displaying acceptable values according to the RMSEA (≤0.080), fell below acceptable values according to the CFI and the TLI (<0.900). The six-factor CFA, in addition to being the only model that displayed adequate values in all indices (i.e., CFI, TLI, and RMSEA), also reported the lowest values in AIC, BIC and ABIC. Then, given that the one-factor CFA and the second-order CFA models fell below acceptable values of fit, and they are considered an essential prerequisite for a psychometrically good model [44], neither of the two solutions were retained for further analysis.

As observed in Table 3, relying on the six-factor CFA model, all specific stressors were generally well-defined by high and significant factor loadings in all items (λ = 0.42 to 0.93, *M* = 0.77). On the other hand, all factor correlations (see Table 4) between the six stressors were significant and positive, with the only exception of the association between ambiguity demands from administration and student diversity (*r* = 0.08, *p* > 0.05). Nonetheless, in line with the above analyses, all correlations between the different teacher stressors were low or moderate (|*r*| = 0.08 to 0.46, *M* = 0.30), offering additional support to the assumption that a multifactor structure seems more adequate when sources of stress are examined.

Starting with the retained six-factor CFA model, we conducted a six-step sequence measurement invariance of the MSS ratings across samples of male and female teachers. As noted in Table 4, results revealed full measurement invariance because none of the six steps fell below the recommended guidelines (ΔCFI and ΔTLI > 0.010; ΔRMSEA ≥ 0.015). In addition, all the measurement invariance models indicated adequate model fit according to the CFI, the TLI, and the RMSEA.

### 3.2. Teacher Stressors and Psychological Functioning

Latent CFA factors representing the three BPNs, job satisfaction, burnout, anxiety, depression, intention to quit the job, and gender, were added to the six-factor CFA model (χ^2^ = 3517.57, df = 1921, *p* < 0.001; CFI = 0.922; TLI = 0.915; RMSEA = 0.038, 90% CI = [0.036–0.040]). As observed in Table 5, all the teacher stressors were significantly and negatively correlated with autonomy satisfaction. However, the significant correlations between stressors and the needs for competence and relatedness were scarcer. In particular, the stressors of student misbehavior, student diversity, and workload were significantly and negatively correlated with competence satisfaction, whereas the stressors of lack of shared decision-making, ambiguous demands from administration, and workload were also significantly and negatively correlated with competence satisfaction. Looking at the teachers’ psychological outcomes, all stressors were significantly and negatively correlated with job satisfaction (with the only exception of insufficient parent involvement, *p* > 0.05), whereas the opposite (i.e., positively) was true for burnout (with the only exception of student misbehavior factor, *p* > 0.05), anxiety, and depression factors. Finally, only the stressors of lack of shared decision-making, ambiguous demands from administration, and workload were significantly and positively correlated with intention to quit the job.

**Figure 1:**
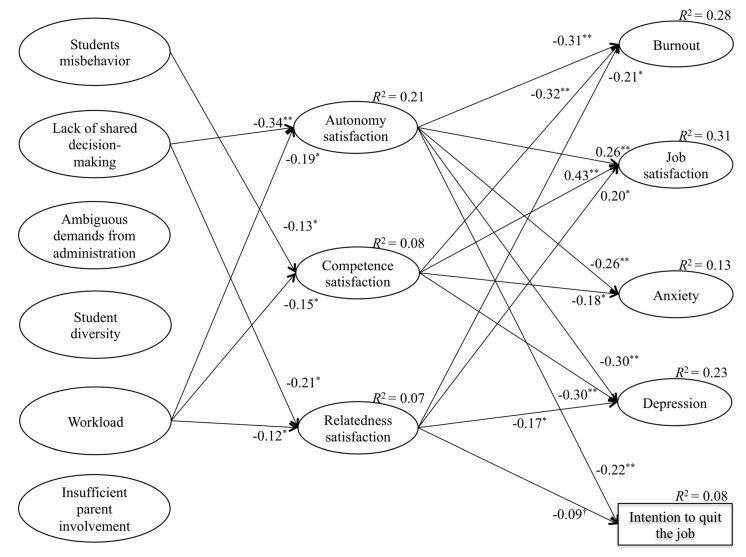
Structural model that relates teachers’ stressors with the satisfaction of the basic psychological needs and teachers’ psychological outcomes. This is a figure, Schemes follow the same formatting. Note. † = *p* < 0.80. * = *p* < 0.05. ** = *p* < 0.01.

In a second step, we relied on an SDT approach to explain the stressor effects, using SEM procedures (χ^2^ = 3769.57, df = 1903, *p* <.001; CFI = 0.910; TLI = 0.901; RMSEA = 0.040, 90% CI = [0.038–0.042]). As observed in Figure 1, the six stressors (i.e., theoretical antecedents) were hypothesized to predict the three BPNs, whereas the three basic needs were hypothesized to predict the rest of the outcomes. Student misbehavior was only significantly negatively related to competence satisfaction. Further, lack of shared decision-making was negatively related to autonomy and relatedness satisfaction, whereas workload was negatively related to the three BPNs. Looking at the BPNs, autonomy satisfaction was significantly and negatively related to burnout, anxiety, depression and intention to quit the job, and significantly and positively related to job satisfaction. Competence and relatedness satisfaction showed the same patterns of associations but with two exceptions. Whereas competence satisfaction was not significantly related to intention to quit the job, relatedness satisfaction was not significantly related to anxiety.

## 4. Discussion

The outcomes of working under stressful conditions in the teaching context have been widely examined to date [1,15,45]. However, little is known about the dimensionality of teacher stressors, cross-gender generalizability of scales, and the relative impact of different stressors on teachers’ psychological functioning. Using the six stressors of the MSS [6], the present study addressed two aims to shed more light on these previous issues.

### 4.1. Dimensional Structure and Generalizability of the Multicontext Stressors Scale

The first aim proposed by this study was to investigate what type of factorial structure should be used to measure teacher stressors, in particular when the MSS is used. Goodness-of-fit indices revealed a statistical superiority of the six-factor structure of the MSS, when compared to both the one-factor structure and the second-order factor structure. In addition, statistical research points out that when a potential global factor remains hidden, factor correlation analysis between specific factors (i.e., stressors) tends to provide high factor correlations, since it is the main way to manifest the existence of cross-loadings [46]. Yet, consistent with prior research [15], our results reported low to moderate correlations between the six stressors of the MSS, indicating—together with the factor loadings—a high specificity of each item on its theoretical specific stressor. As a theoretical contribution, this study expands research on the factorial structure of the MSS, investigating the fit of both the single- and multifactor- structures, and covering one of the main limitations of the previous studies conducted with this scale [6,15]. In line with findings from other recent studies [17,18], the results of the present study not only support our assumption based on the fact that sources of stress should be measured separately in teachers, but they also question results of previous research that examined teachers’ stressful conditions via one single common stressor [6,45].

On the other hand, this study also aimed to analyze the extent to which the factorial structure of MSS ratings remains invariant across samples of male and female teachers. The results of the present study support the complete invariance across gender of the six-factor structure of the MSS, a gap which had not been previously investigated [6,15]. Importantly, the measurement invariance across gender had not been previously tested, either, in past studies that had investigated gender effects on teacher stressors using other scales [20]. Therefore, our results could represent a relevant advance towards more adequately measuring stressors within the teaching context. In addition, these results represent a methodological contribution, demonstrating that the psychometric properties of MSS scores generalize to significant subgroups of teachers (i.e., males and females) [20], which is a key requirement of a good psychometric validation study [19].

### 4.2. Teacher Stressors and Psychological Functioning

The second aim of this study was to investigate how the six stressors of the MSS may differentially predict the three BPNs, as well as to examine the relationships between basic needs and teachers’ psychological outcomes. According to the tenets of SDT [11,12,13], the SEM results of this study revealed negative relationships between three of the six teacher stressors and BPNs. First, student misbehavior was negatively related to competence satisfaction. A possible explanation is that feelings of stress linked to students’ misbehavior, such as amotivation, lack of interest, or lack of discipline, could interfere in perceptions that teachers have of their performance and ability (i.e., competence satisfaction) [9]. In support of this possible explanation, a recent qualitative study pointed out that students’ disruptive behaviors made teaching notably difficult to conduct [7]. Second, a lack of shared decision-making was negatively related to autonomy and relatedness satisfaction. For teachers to fully experience a sense of autonomy satisfaction, it is crucial that they feel a sense of psychological freedom and meaningfulness to make own teaching decisions [12]. Therefore, the scarce organizational flexibility of schools and administration, and inability to participate in the school board decisions could explain the negative relationship between feelings of lack of shared decision-making and teachers’ autonomy satisfaction. Likewise, teachers’ relatedness is nurtured when they feel connected not only to other teachers, but also to their superiors, giving rise to a warmer and closer working environment in schools [47]. So, difficulties in interacting with principals and administration could create interaction social barriers in the teaching context, providing a possible explanation for the negative relationship between this stressor and the need for relatedness [48]. Third, workload was negatively related to the three BPNs. In agreement with our results, 85% of the teachers who participated in a qualitative study identified workload as one of the most detrimental factors for their well-functioning at work [7]. High workload could lead teachers to perform tasks in a more mechanical way, unconsciously reducing their decision-making (i.e., autonomy satisfaction) [49]. Likewise, the large number of obligations, such as preparing lessons, assessments, meetings, or extracurricular activities could lead to the inadequate preparation of some tasks, affecting the teachers’ competence satisfaction [7]. Similarly, other factors such as that school environment allow little time for teachers to share school experiences and interpersonal problems with fellow teachers, which contributes to the lack of a sense of connection among teachers (i.e., relatedness satisfaction) [7].

However, SEM results showed that the ambiguity of administration, student diversity, and insufficient parent involvement did not predict BPN satisfaction. Although more research seems required, some interpretations may help to explain these results. Studies conducted two decades ago [4,50], indicated that teachers relied more on their peers than school administration to keep informed of administrative-related tasks. Yet, online social networking tools and emerging technological progress in recent years may have cushioned the stressful effect of administration on teachers’ psychological functioning. Student diversity was added to the MSS with the aim of identifying racial and cultural heterogeneity effects in the classrooms, due to the emerging immigration phenomenon in Spain during the past decade [6,15]. However, stressors are not static factors. In this light, teachers could have learned to successfully cope with student diversity, reducing the impact on their psychological functioning at work. Finally, one past interview-based study identified insufficient parent involvement as a relevant stressor among primary school teachers [2]. However, the teachers in this study dealt with older students (i.e., secondary school students), who, therefore, also had a higher sense of autonomy. This fact could explain why teachers of the present study did not perceive the parents’ involvement as a stress factor to hinder their BPNs.

On the other hand, we expected to find positive relationships between the three BPNs and job satisfaction, whereas the opposite was suggested for burnout, anxiety, depression, and intention to quit the job. Consistent with previous research, the three BPNs were positively related to job satisfaction (e.g., burnout, anxiety) [25,26], and negatively related to most maladaptive teacher outcomes [27,28,29,30]. These results broadly support the role of BPN satisfaction in the teaching context, not only to experience well-functioning at work (i.e., job satisfaction), but also to buffer the emergence of negative outcomes. As a practical contribution, school policymakers should focus their efforts on the design and application of stressor prevention strategies, being especially wary of student misbehavior, lack of teachers’ capacity to participate in shared decision-making, and heavy workload. Finally, it is important to note that the teacher stressors of the MSS showed different association patterns with the BPNs. Consistent with Skaalvik and Skaalvik’s findings [17,18], these results support our hypothesis on stressor multidimensionality, highlighting that stressful teaching conditions should be analyzed as individual factors.

### 4.3. Limitations and Directions for Future Research

The first limitation is the low response rate (i.e., 8%) of the study sample. Teacher participation was voluntary, so there was no random choice (i.e., intentional) involved to ensure that the sample was representative of this group. Consequently, caution is needed when generalizing these results. Similarly, this study was conducted in a sample of secondary school teachers, which limits the generalizability of the findings to other education levels (i.e., pre-elementary and elementary education). Future studies should replicate the design of this study with a more representative sample, and with teachers belonging to other educational stages. The second limitation is related to the cross-sectional design. Even though the SEM analyses are based on a theoretical assumption of causality (i.e., as posited in the SDT framework), longitudinal designs are required to ensure stronger causal conclusions. The third limitation is related to the assessment of intention to quit the job, which was based only on a dichotomous answer. Future studies should consider a more complete measurement of intention to quit the job. Last, the present study extends previous research by investigating the extent to which the six MSS stressors are associated with BPN satisfaction. Future qualitative studies should be conducted to provide a further explanation of the relationship of these six stressors and teachers´ BPNs. Moreover, examining the relationship of these six stressors with the "dark-side" of the SDT (i.e., basic needs frustration) may also be a new avenue of research.

## 5. Conclusions

The present study expands previous research conducted with the MSS [6,15] by providing evidence of better psychometric properties of the six-factor structure compared to the one-factor structure, as well as by providing evidence on complete measurement invariance across gender of the MSS. Based on these findings, studies aimed at examining the sources of stress in teachers should be performed separately and never rely on a single common stressor. In addition, because teacher stressors could be perceived differently by males and females, it seems advisable to rely on invariant scales, such as the MSS. On the other hand, the present study also shows that the three BPNs were only predicted by three teacher stressors of the MSS. Whereas the needs for autonomy and relatedness are hindered by feelings of lack of shared decision-making and workload, the need for competence is interfered by the feelings of student misbehavior and workload. Given the importance of the influence of BPNs on teachers’ psychological functioning, boosting teachers’ strategies for coping with these sources of stress should be a priority for school leaders and the teachers themselves.

## Figures and Tables

**Table 1:** Descriptive statistics and Pearson’s correlations for the items of the Multicontext Stressors Scale.

Items	*M (SD)*	1	2	3	4	5	6	7	8	9	10	11	12	13	14	15	16	17	18	19	20	21	22	23	24	25	26	27	28	29	30	31
SMB 1	1.79 (0.73)	-																														
SMB 2	1.18 (0.80)	.43	-																													
SMB 3	1.93 (0.75)	.64	.38	-																												
SMB 4	1.95 (0.76)	.62	.35	.85	-																											
SMB 5	1.86 (0.77)	.63	.36	.78	.82	-																										
SMB 6	1.84 (0.74)	.57	.30	.73	.77	.81	-																									
SMB 7	2.00 (0.85)	.35	.46	.42	.40	.40	.40	-																								
LMD 8	1.10 (0.88)	.12	.30	.17	.14	.17	.16	.31	-																							
LMD 9	1.09 (0.86)	.*03*	.23	.09	.*06*	.10	.09	.17	.60	-																						
LMD 10	1.06 (0.93)	.*05*	.25	.11	.09	.13	.11	.21	.54	.68	-																					
LMD 11	1.00 (0.90)	.*03*	.21	.09	.09	.14	.11	.20	.51	.65	.65	-																				
LMD 12	1.10 (0.92)	.08	.26	.14	.13	.17	.15	.23	.46	.57	.52	.66	-																			
ADA 13	1.86 (1.00)	.*06*	.17	.16	.15	.18	.19	.17	.32	.33	.42	.32	.35	-																		
ADA 14	1.96 (0.94)	.10	.22	.19	.18	.21	.21	.24	.30	.33	.43	.36	.37	.74	-																	
ADA 15	1.90 (1.00)	.*06*	.16	.14	.12	.16	.18	.16	.25	.29	.39	.29	.28	.79	.76	-																
ADA 16	2.01 (0.95)	.13	.22	.19	.19	.21	.25	.22	.27	.25	.34	.28	.29	.75	.74	.82	-															
ADA 17	2.12 (0.93)	.11	.16	.17	.20	.20	.22	.24	.25	.26	.28	.24	.25	.62	.61	.65	.67	-														
ADA 18	2.14 (0.92)	.14	.18	.16	.19	.21	.21	.27	.26	.23	.28	.23	.22	.61	.62	.65	.67	.87	-													
SDV 19	1.47 (0.82)	.19	.15	.30	.30	.28	.28	.22	.15	.08	.*03*	.04	.*07*	.*05*	.04	.*07*	.*05*	.11	.11	-												
SDV 20	1.41 (0.80)	.18	.17	.29	.29	.25	.25	.23	.17	.08	.*05*	.*07*	.09	.09	.08	.08	.*06*	.13	.15	.86	-											
SDV 21	0.72 (0.74)	.16	.13	.23	.22	.21	.18	.17	.14	.09	.*04*	.09	.09	.*03*	.*00*	.*01*	.*04*	.*03*	.*06*	.40	.40	-										
SDV 22	1.05 (0.71)	.15	.17	.26	.29	.27	.24	.20	.13	.11	.*04*	.08	.11	.*02*	.*04*	-.*01*	.*05*	.08	.10	.59	.60	.59	-									
SDV 23	1.01 (0.81)	.16	.18	.24	.26	.24	.21	.20	.10	.*01*	-.*04*	.*05*	.*01*	.*02*	.08	.*04*	.08	.*02*	.*04*	.56	.57	.51	.62	-								
WL 24	1.83 (0.92)	.18	.17	.28	.31	.26	.27	.21	.21	.22	.19	.17	.21	.26	.28	.26	.33	.28	.31	.24	.29	.13	.23	.29	-							
WL 25	1.91 (0.93)	.16	.19	.25	.29	.24	.22	.18	.23	.24	.24	.20	.21	.31	.37	.30	.38	.39	.38	.18	.22	.10	.15	.22	.70	-						
WL 26	1.83 (0.95)	.13	.21	.23	.28	.24	.25	.20	.22	.28	.24	.27	.27	.32	.39	.31	.37	.42	.41	.19	.24	.13	.18	.22	.60	.81	-					
WL 27	1.71 (0.95)	.15	.25	.19	.18	.19	.20	.23	.31	.33	.31	.30	.29	.36	.40	.32	.38	.38	.39	.20	.26	.11	.21	.18	.51	.66	.64	-				
IPI 28	1.45 (0.84)	.20	.27	.30	.32	.30	.33	.31	.21	.26	.24	.24	.25	.22	.25	.23	.29	.26	.28	.23	.25	.21	.23	.22	.23	.23	.25	.27	-			
IPI 29	1.39 (0.80)	.19	.26	.29	.29	.29	.33	.31	.19	.22	.21	.17	.20	.22	.28	.28	.28	.29	.29	.19	.21	.18	.21	.18	.19	.22	.22	.24	.88	-		
IPI 30	1.11 (0.88)	.23	.42	.24	.25	.27	.24	.35	.32	.24	.22	.22	.32	.20	.28	.22	.25	.25	.24	.13	.18	.14	.15	.14	.21	.28	.30	.28	.51	.53	-	
IPI 31	1.07 (0.74)	.23	.29	.21	.22	.22	.22	.30	.21	.30	.26	.29	.31	.21	.23	.22	.19	.17	.15	.15	.17	.13	.14	.18	.20	.21	.24	.21	.54	.54	.48	-

Note: M = Means; SD = Standard Deviation; SMB = Student misbehavior; LMD = Lack of shared decision-making; ADA = Ambiguous demands from administration; SDV = Student diversity; WL = Workload; IPI = Insufficient parent involvement. Items correlations ≥ .08 and ≥ .11 are significant at the level *p* < 0.05 and *p* < 0.01 respectively; Non significant correlations are marked in italics.

**Table 2:** Goodness-of-Fit Statistics.

Model	χ^2^	df	CFI	TLI	RMSEA [90% CI]	AIC	BIC	ABIC
One-factor CFA	7649.49 *	434	0.321	0.273	0.169 [0.165–0.172]	41,249.12	41,655.52	41,360.28
Six-factor CFA	1083.57 *	419	0.937	0.930	0.052 [0.049–0.056]	33,619.16	34,104.22	33,751.84
Second-order factor CFA	1702.05 *	428	0.880	0.870	0.071 [0.068–0.075]	34,298.28	34,730.90	34,416.61

Note: CFA = Confirmatory factor analysis; df = Degrees of freedom; CFI = comparative fit index; TLI = Tucker-Lewis index; RMSEA = root mean square error of approximation; CI = confidence interval; AIC = Akaike information criterion; BIC = Bayesian information criterion; ABIC = Sample size adjusted BIC. * *p* < 0.01.

**Table 3:** Standardized Factor Loadings (λ) and Uniquenesses (δ) for the six-factor CFA model of the MSS.

Items	λ	δ
***Student misbehavior (SMB)***		
1. The students “couldn’t-care-less” attitude.	0.69 **	0.52
2. Student pressure on teachers.	0.42 **	0.83
3. Students’ demotivation	0.90 **	0.20
4. Students’ lack of interest.	0.93 **	0.14
5. Students’ idleness.	0.90 **	0.20
6. Students not getting involved.	0.84 **	0.29
7. Students’ lack of discipline.	0.47 **	0.77
***Lack of shared decision-making (LDM)***		
8. The impositions of my superiors (Headmaster, Head of Department, Inspections, etc.).	0.67 **	0.55
9. The organisational inflexibility of the institution and departments.	0.82 **	0.32
10. The lack of definition of the institution’s educational policy.	0.79 **	0.37
11. The fact that it is not possible to take part in decision-making.	0.81 **	0.34
12. The lack of autonomy to make my own decisions.	0.72 **	0.48
***Ambiguous demands from administration (ADA)***		
13. The ambiguity of the administration’s educational policy.	0.86 **	0.25
14. The indifference on the administration’s part to school-related problems.	0.84 **	0.29
15. The lack of definition of the administration’s educational policy.	0.91 **	0.18
16. The contradictory demands we receive from the administration.	0.89 **	0.22
17. The frequent changes to the study curriculum.	0.73 **	0.46
18. The frequent legal changes concerning matters of education.	0.73 **	0.46
***Student diversity (SDV)***		
19. The diversity in student’s paces of learning.	0.91 **	0.17
20. The diversity in the levels of students’ knowledge.	0.92 **	0.16
21. The cultural and racial diversity among students.	0.49 **	0.76
22. The diversity of students’ learning styles.	0.69 **	0.53
23. Students’ heterogeneity in class.	0.65 **	0.57
***Workload (WL)***		
24. Lack of time.	0.73 **	0.46
25. Work overload.	0.92 **	0.16
26. Excessive academic load.	0.86 **	0.25
27. Difficulty to combine teaching with other roles or tasks that are expected of you.	0.73 **	0.46
***Insufficient parent involvement (IPI)***		
28. Parents’ collaboration is insufficient.	0.93 **	0.14
29. Parents are not involved enough.	0.93 **	0.14
30. Pressure from parents.	0.57 **	0.67
31. Parents are not informed enough.	0.59 **	0.65

Note: * *p* < 0.05. ** *p* < 0.01.

**Table 4:** Measurement Invariance across Gender for the six-factor CFA Model.

Invariance	χ^2^ (df)	CFI	TLI	RMSEA [90% CI]	CM	ΔCFI	ΔTLI	ΔRMSEA
Configural (C)	1592.50 * (838)	0.930	0.922	0.056 [0.052–0.060]	-	-	-	-
Weak (W)	2002.76 * (882)	0.928	0.924	0.068 [0.064–0.072]	(C)	−0.002	+0.002	+0.012
Strong (S)	2054.36 * (925)	0.921	0.916	0.070 [0.066–0.075]	(W)	−0.007	−0.008	+0.002
Strict (ST)	2023.52 * (940)	0.913	0.908	0.071 [0.066–0.076]	(S)	−0.008	−0.008	+0.001
Var.-cov. (VC)	4042.93 * (946)	0.904	0.902	0.063 [0.059–0.066]	(ST)	−0.009	−0.006	+0.008
Latent mean	5485.69 * (975)	0.900	0.901	0.063 [0.059–0.067]	(VC)	−0.004	−0.001	0.000

Note: *χ*^2^ = Scaled chi-square test of exact fit; df = Degrees of freedom; CFI = Comparative fit index; TLI = Tucker-Lewis index; RMSEA = Root mean square error of approximation; 90% CI = 90% Confidence interval of the RMSEA; CM = Comparison model; Δ = Change in fit information relative to the CM; Var.-cov. = Variance-covariance; * *p* < 0.01.

**Table 5:** Latent Correlations between Stressors, Basic Psychological Needs and Teachers’ Psychological Outcomes.

	*M* (*SD*)	1. SMB	2. LDM	3. ADA	4. SDV	5. WL	6. PII	7. A	8. C	9. R	10. JS	11. BUR	12. ANX	13. DP	14. IQ
1. Stu. misbehavior (SMB)	1.80 (0.60)	-													
2. Lack of decision (LDM)	1.06 (0.73)	.18 **	-												
3. Ambiguous adm. (ADA)	1.99 (0.82)	.23 **	.46 **	-											
4. Stu. diversity (SDV)	1.16 (0.63)	.39 **	.11 *	.08	-										
5. Workload (WL)	1.82 (0.80)	.33 **	.36 **	.46 **	.30 **	-									
6. Ins. parents involv. (IPI)	1.25 (0.67)	.38 **	.32 **	.33 **	.30 **	.30 **	-								
7. Autonomy (A)	4.74 (0.82)	-.13 *	-.37 **	-.24 **	-.19 **	-.30 **	-.12 *	-							
8. Competence (C)	5.00 (0.60)	-.14 *	-.02	-.08	-.14 *	-.16 *	-.08	.50 **	-						
9. Relatedness (R)	4.58 (0.91)	-.01	-.20 **	-.10 *	-.02	-.14 *	-.05	.40 **	.35 **	-					
10. Job satisfaction (JS)	4.25 (1.09)	-.15 *	-.14 *	-.18 **	-.15 *	-.24 **	-.09	.46 **	.56 **	.37 **	-				
11. Burnout (BUR)	2.71 (0.87)	.09	.28 **	.17 *	.17 *	.23 **	.16 *	-.49 **	-.50 **	-.37 **	-.77 **	-			
12. Anxiety (ANX)	1.03 (0.51)	.21 **	.24 **	.28 **	.16 *	.38 **	.16 *	-.34 **	-.30 **	-.21 **	-.35 **	.31 **	-		
13. Depression (DP)	0.56 (0.46)	.22 **	.28 **	.25 **	.15 *	.33 **	.17 *	-.44 **	-.47 **	-.34 **	-.60 **	.61 **	.69 **	-	
14. Intention to quit (IQ)	-	.06	.18 **	.17 **	.08	.26 **	.02	-.26 **	-.18 **	-.17 **	-.43 **	.48 **	.26 **	.36 **	-

Note: Lack of intention to quit the job scored 0 and intention to quit the job scored 1; * *p* < 0.05. ** *p* < 0.01.
